# M1 and M2 Monocytes in Rheumatoid Arthritis: A Contribution of Imbalance of M1/M2 Monocytes to Osteoclastogenesis

**DOI:** 10.3389/fimmu.2017.01958

**Published:** 2018-01-08

**Authors:** Shoichi Fukui, Naoki Iwamoto, Ayuko Takatani, Takashi Igawa, Toshimasa Shimizu, Masataka Umeda, Ayako Nishino, Yoshiro Horai, Yasuko Hirai, Tomohiro Koga, Shin-ya Kawashiri, Mami Tamai, Kunihiro Ichinose, Hideki Nakamura, Tomoki Origuchi, Ritsuko Masuyama, Kosuke Kosai, Katsunori Yanagihara, Atsushi Kawakami

**Affiliations:** ^1^Department of Immunology and Rheumatology, Nagasaki University Graduate School of Biomedical Sciences, Nagasaki, Japan; ^2^Department of Community Medicine, Nagasaki University Graduate School of Biomedical Sciences, Nagasaki, Japan; ^3^Medical Education Development Center, Nagasaki University Hospital, Nagasaki, Japan; ^4^Center for Comprehensive Community Care Education Medicine, Nagasaki University Graduate School of Biomedical Sciences, Nagasaki, Japan; ^5^Department of Rheumatology, Clinical Research Center, NHO Nagasaki Medical Center, Omura, Nagasaki, Japan; ^6^Center for Bioinformatics and Molecular Medicine, Nagasaki University Graduate School of Biomedical Sciences, Nagasaki, Japan; ^7^Department of Rehabilitation Sciences, Nagasaki University Graduate School of Biomedical Sciences, Nagasaki, Japan; ^8^Department of Molecular Bone Biology, Nagasaki University Graduate School of Biomedical Sciences, Nagasaki, Japan; ^9^Department of Laboratory Medicine, Nagasaki University Graduate School of Biomedical Sciences, Nagasaki, Japan

**Keywords:** rheumatoid arthritis, monocytes, anticitrullinated protein antibody, osteoclasts, inflammation

## Abstract

**Objectives:**

We investigated the relationships among M1 monocytes, M2 monocytes, osteoclast (OC) differentiation ability, and clinical characteristics in patients with rheumatoid arthritis (RA).

**Methods:**

Peripheral blood mononuclear cells (PBMCs) were isolated from RA patients and healthy donors, and we then investigated the number of M1 monocytes or M2 monocytes by fluorescence-activated cell sorting. We also obtained and cultured CD14-positive cells from PBMCs from RA patients and healthy donors to investigate OC differentiation *in vitro*.

**Results:**

Forty RA patients and 20 healthy donors were included. Twenty-two patients (55%) were anticitrullinated protein antibody (ACPA) positive. The median M1/M2 ratio was 0.59 (0.31–1.11, interquartile range). There were no significant differences between the RA patients and healthy donors. There was a positive correlation between the M1/M2 ratio and the differentiated OC number *in vitro* in RA patients (ρ = 0.81, *p* < 0.001). The ACPA-positive patients had significantly higher M1/M2 ratios *in vivo* (*p* = 0.028) and significantly greater numbers of OCs *in vitro* (*p* = 0.005) than the ACPA-negative patients. Multivariable regression analysis revealed that the M1/M2 ratio was the sole significant contribution factor to *in vitro* osteoclastogenesis. RA patients with M1/M2 ratios >1 (having relatively more M1 monocytes) had higher C-reactive protein and erythrocyte sedimentation rates than RA patients with M1/M2 ratios ≤1. M1-dominant monocytes *in vitro* produced higher concentrations of interleukin-6 upon stimulation with lipopolysaccharide than M2 monocytes.

**Conclusion:**

M1/M2 monocytes imbalance strongly contributes to osteoclastogenesis of RA patients. Our findings cast M1 and M2 monocyte subsets in a new light as a new target of treatments for RA to prevent progression of osteoclastic bone destruction.

## Introduction

Rheumatoid arthritis (RA) is the most common form of inflammatory arthritis and is characterized by inflammation and matrix destruction of bone and cartilage (1). RA affects approximately 1% of the world’s population (2). Bone resorption (i.e., bone erosion, a hallmark of RA) was demonstrated histopathologically to be caused mainly by osteoclasts (OCs) (3). OCs are differentiated from monocytes and macrophages through the stimulation of receptor activator for nuclear factor kappa-B ligand (RANKL) in the presence of macrophage colony-stimulating factor (M-CSF) (4). Increased *in vitro* osteoclastogenesis was observed in RA patients (5).

Monocytes can differentiate into proinflammatory, microbicidal M1 macrophage, or anti-inflammatory M2 macrophage subtypes (6). The concept of M1 macrophages and M2 macrophages was formulated by mirroring the Th1/Th2 polarization concept (7). Macrophages are activated toward M1 macrophages by infectious microorganism-related molecules such as lipopolysaccharides (LPSs) and by inflammatory cytokines such as interferon-γ (7). M1 macrophages can produce toxic effector molecules such as reactive oxygen species and nitric monoxide, and inflammatory cytokines such as interleukin (IL)-1β, tumor necrosis factor (TNF), and IL-6 (8). Conversely, M2 macrophage polarization is observed in response to Th2-related cytokines such as IL-4 and IL-13 (9). Anti-inflammatory cytokines such as IL-10 and TGF-β are also associated with M2 macrophage polarization (10).

There is growing evidence that an imbalance of M1 macrophages and M2 macrophages is associated with many diseases such as asthma (11), chronic obstructive pulmonary disease (12), atherosclerosis (13) and tumors (14). Interestingly, in addition to macrophages, regarding monocyte subsets, M1 monocytes and M2 monocytes mirroring the M1/M2 macrophage polarization concept were suggested, and they are reported to be associated with diabetes mellitus (15) and hypercholesterolemia and atherosclerosis (16). These M1/M2 monocyte subsets are different from the traditional monocyte subsets defined by CD14 and CD16 expression (17). However, the roles of this novel concept of monocytes in the etiology of various diseases have not been elucidated. Moreover, little is known regarding the relationships between osteoclastogenesis and M1/M2 monocyte subsets.

In this study, we investigated the relationships among M1/M2 monocyte subsets and the differentiation ability of OCs, which are derived from monocytes. We also sought to elucidate the relationships among M1 and M2 monocyte subsets, osteoclastogenesis, and the clinical backgrounds of RA patients.

## Materials and Methods

### Patients

We enrolled 40 patients with RA who were followed at Nagasaki University Hospital between January and July 2016. Their RA was diagnosed based on the ACR/EULAR 2010 RA classification criteria (18). We collected demographic, clinical, and laboratory characteristics of the RA patients and treatments for RA at the time of the monocyte subset analyses. We also examined the patients’ clinical disease activities of RA as disease activity score (DAS) 28-erythrocyte sedimentation rate (ESR) and DAS 28-C reactive protein (CRP) at the time of the monocyte subset analyses. We defined the rheumatoid factor (RF)-positive status as >15.0 U/mL (upper normal value, measured by a latex-enhanced immunonephelometric assay; Dade Behring, Marburg, Germany).

We also defined the anticitrullinated protein antibody (ACPA)-positive status as >4.5 U/mL [upper normal value, measured by enzyme-linked immunosorbent assay (ELISA); DIASTAT Anti-CCP; Axis-Shield, Dundee, UK].

We evaluated the radiographic severity of peripheral joint damage at entry according to Steinbrocker stage (19). In addition, we defined Steinbrocker stage I or II as non-erosive disease and Steinbrocker stage III or IV as erosive disease.

The patients’ characteristics are summarized in Table 1. We also enrolled 20 healthy donors to compare to the RA patients. This study was performed in accordance with the Declaration of Helsinki and was approved by the Investigation and Ethics Committee at Nagasaki University Hospital (approval number: 12072361). Patients and healthy donors gave their informed consent to be subjected to the protocol.

**Table 1:** Demographic, clinical, and laboratorial characteristics, treatments and disease activities of total 40 RA patients.

Characteristics	Number
Females, *n* (%)	31 (78)
Age at entry (years), median (IQR)	63 (49–77)
Disease duration (years), median (IQR)	3.0 (1.0–15.0)
Rheumatoid factor positive, *n* (%)	28 (70)
ACPA-positive, *n* (%)	22 (55)
Tender 28-joint count, median (IQR)	1 (0–5)
Swollen 28-joint count, median (IQR)	0 (0–2)
ESR (mm/h), median (IQR)	18 (8–36)
CRP (mg/dL), median (IQR)	0.12 (0.02–0.55)
PtGA, VAS 0–100 mm, median (IQR)	20 (11–55)
DAS28-ESR	3.0 (1.9–4.2)
DAS28-CRP	2.2 (1.5–3.5)
Concomitant MTX use, *n* (%), dose (mg/week)	18 (45), 5 (4–6)
Concomitant prednisolone use, *n* (%), dose (mg/day)	22 (55), 8 (6–10)
Biologics, *n* (%)	17 (43) (7 TNF inhibitors, 5 TCZ, 5 ABT)
Steinbrocker stage	I: 23, II: 4, III: 3, IV:10
Erosive disease (Steinbrocker class: III or IV), *n* (%)	13 (33)

*IQR, interquartile range; ACPA, anticitrullinated protein antibody; ESR, erythrocyte sedimentation rate; CRP, C-reactive protein; PtGA, patient global assessment; VAS, visual analog scale; DAS, disease activity score; MTX, methotrexate; TNF, tumor necrosis factor; TCZ, tocilizumab; ABT, abatacept*.

### Fluorescence-Activated Cell Sorting

For the identification and quantification of circulating monocyte subsets in whole peripheral blood samples, we performed fluorescence-activated cell sorting (i.e., a FACS analysis). Peripheral blood mononuclear cells (PBMCs) were isolated from whole blood using Lymphoprep (Cosmo Bio, Tokyo). PBMCs were stained for the examination of the expression of CD14, CD68, CCR2, CX3CR1, CD163, and CD206 using PE-conjugated anti-CD14 (BD Biosciences, San Jose, CA, USA), FITC-conjugated anti-CD68 (DakoCytomation, Carpinteria, CA, USA), Alexa Fluor 647-conjugated anti-CCR2 (BD Biosciences), FITC-conjugated anti-CX3CR1 (BioLegend, San Diego, CA, USA), and Alexa Fluor 647-conjugated anti-CD163 and APC-conjugated anti-CD206 (all from BD Biosciences) monoclonal antibodies. FITC-conjugated mouse IgG2b, FITC-conjugated rat IgG2b, AlexaFluor647-conjugated mouse IgG2b, AlexaFluor647-conjugated mouse IgG1 and APC-conjugated mouse IgG1 (all from BD Biosciences) were used isotype controls.

**Figure 1:**
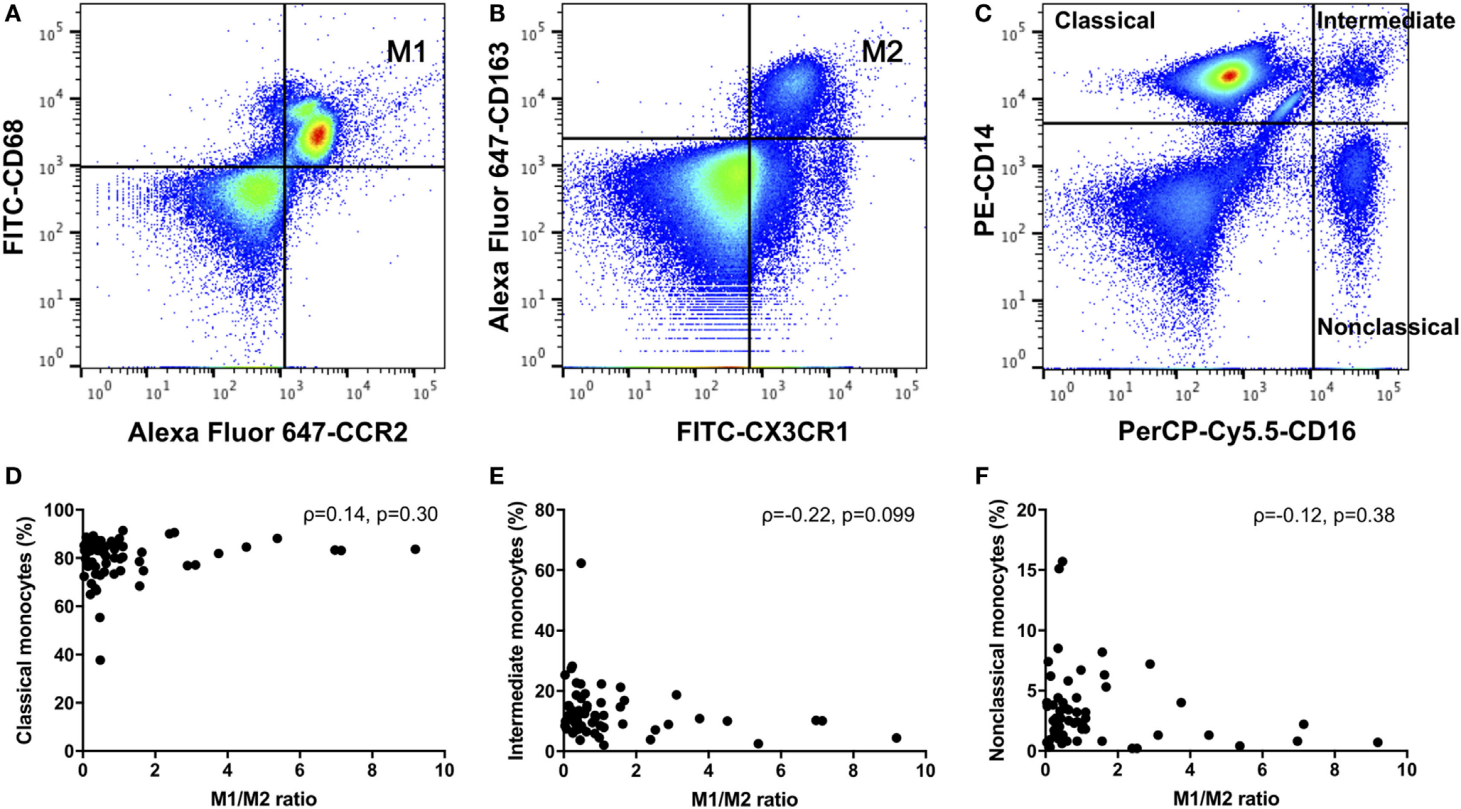
**(A)** M1 monocytes were defined as positive for CD14, CD68, and CCR2. **(B)** M2 monocytes were defined as positive for CD14, CX3CR1, and CD163 monocytes were defined as M2 monocytes. **(C)** We quantified three types of monocytes: classical CD14^++^CD16^−^, intermediate CD14^++^CD16^+^, and nonclassical CD14^+^CD16^+^ monocytes. There were no correlations between the M1/M2 ratio and the **(D)** classical, **(E)** intermediate, or **(F)** nonclassical monocytes.

We defined positive CD14, CD68, and CCR2 monocytes as M1 monocytes (Figure 1A), and in separate tubes, we defined positive CD14, CX3CR1, and CD163 or CD206 monocytes as M2 monocytes (Figure 1B). First, we gated monocytes in a forward scatter/sideward scatter (FSC/SSC) dot-plot. Second, we gated CD14-positive monocytes within monocytes. Finally, we gated M1 or M2 monocytes using isotype controls within CD14-positive monocytes. At the same time, PBMCs were stained with a PE-conjugated anti-CD14 and a PerCP-Cy5.5-conjugated anti-CD16 (BD Biosciences) monoclonal antibodies. We also quantified three types of monocytes: classical CD14^++^CD16^−^, intermediate CD14^++^CD16^+^, and nonclassical CD14^+^CD16^+^ monocytes (Figure 1C) (17).

The relative frequency of these monocyte subsets is expressed as percent within the CD14-positive cell gate. Experiments were performed using a FACS Canto II Flow Cytometer and FACS Diva software (BD Biosciences).

### Osteoclastogenesis

At the same time as the FACS analysis, we purified CD14-positive cells from whole peripheral blood from the RA patients and healthy donors using CD14 M-pluriBead (pluriSelect, Spring Valley, CA, USA). We cultured CD14-positive cells (1 × 10^5^ cells per well) in a 96-well dish with Octeoclast Precursor BulletKit™ Medium containing l-glutamine, penicillin/streptomycin, M-CSF, and RANKL (Lonza, Walkersville, MD, USA) so that the cells would differentiate to OCs. We changed the medium three times a week. At day 12, the cells were fixed in 4% paraformaldehyde and stained with tartrate-resistant acid phosphatase (TRAP) using a TRAP staining kit (Cosmo Bio).

We defined OCs as TRAP staining-positive cells with >3 nuclei. We counted the OCs in the whole wells. Three wells were used to count OCs from one patient or one healthy donor, and we defined the average of the numbers of OCs of three wells as “the numbers of OCs.”

### Pit Formation Assay

Pit formation assays were performed using Dentin Slice (from Ivory, Thin Type for pit formation assay, Wako Pure Chemical Industries, Osaka, Japan.). CD14-positive monocytes were cultured with Octeoclast Precursor BulletKit™ Medium on the dentin slices for 17 days. Resorbed lacunae were visualized by Mayer’s hemalum solution (Merck Millipore, Darmstadt, Germany) staining, and the relative resorbed area was measured under a microscope (BZ-9000, Keyence, Tokyo). Resorbing lacunae were quantified using Image J software (US National Institutes of Health, Bethesda, MD, USA). The relative area of resorbing lacunae is expressed as the percent of the area to the entire dentin slice.

### Stimulation Assays and ELISA

In another set of experiments, CD14+ CX3CR1+ CD163+ (M2 monocyte) cells were isolated from PBMCs derived from healthy donors by FACS. We used the rest of the CD14-positive cells as M1-dominant monocytes because M1 monocytes could not be isolated by FACS due to the intracellular expression of CD68. They were incubated in MEM Alpha medium with 10% fetal bovine serum, 1% penicillin/streptomycin (all from Gibco, Basel, Switzerland) for 24 h in a 12-well dish at 5 × 10^4^ cells per well. Cells were stimulated for 24 h with LPS (100 ng/mL; Sigma-Aldrich, St. Louis, MO, USA) or phosphate-buffered saline as a control, and supernatants were obtained. Proteins in the cultured supernatants were detected by ELISA using an IL-6-specific ELISA kit according to the manufacturer’s instructions (R&D Systems, Minneapolis, MN, USA). Absorption was measured at 450 nm.

### Statistical Analysis

Variables are described using frequencies for categorical variables and with the median and interquartile range (IQR) for quantitative variables. We assessed the association between variables using Wilcoxon’s rank sum test for quantitative variables. Correlations were analyzed using Spearman’s correlation coefficient. Univariate and multivariable regression model analyses were performed to determine the factors that contribute to the number of OCs. All tests were two-sided, and a *p*-value < 0.05 was considered significant. All statistical analyses were performed using JMP Statistical Software, ver. 11 (SAS Institute, Cary, NC, USA) and GraphPad Prism ver. 7.0 (GraphPad Software, San Diego, CA, USA).

## Results

### The Monocyte Subsets (Including the M1/M2 Ratio) Showed No Difference between the RA Patients and Healthy Donors

As shown in Table 2, we compared RA patients with healthy donors for each monocyte subset. The population of CD206-positive M2 monocytes was too small to examine (0–0.5% within CD14-positive cells). Therefore, we redefined positive CD14, CX3CR1 and CD163 monocytes as M2 monocytes (Figure 1B). The median M1 and M2 monocyte percentages in CD14-positive cells were 16.7 and 40.0% in the RA group and 25.8 and 29.7% in the healthy donor group, respectively, with no significant differences between the RA and healthy groups. The RA and healthy groups had nearly equal M1/M2 ratios (0.60 vs. 0.62, respectively; *p* = 0.88). Likewise, the distribution of monocyte subtypes divided by CD14 and CD16 expression showed no significant differences between the RA patients and healthy donors. There was no correlation between the M1/M2 ratio and these subsets (Figures 1D–F).

**Table 2:** Monocyte subsets and osteoclastogenesis of RA patients and healthy donors.

Monocyte subset	RA patients, *n* = 40	Healthy donors, *n* = 20	*p*-Value
Classical monocytes (%),^a^ median (IQR)	82.6 (76.4–86.5)	82.4 (74.7–85.6)	0.77
Intermediate monocytes (%),^a^ median (IQR)	11.0 (8.5–14.9)	10.1 (7.4–16.8)	0.99
Nonclassical monocytes (%),^a^ median (IQR)	2.4 (1.1–4.0)	2.5 (0.9–4.4)	0.76
M1 monocytes (%),^a^ median (IQR)	16.7 (6.6–40.1)	25.8 (11.0–43.9)	0.44
CD163-positive M2 monocytes (%),^a^ median (IQR)	40.0 (11.9–67.1)	29.7 (23.1–68.2)	0.83
CD206-positive M2 monocytes (%),^a^ median (IQR)	0 (0–0.1)	0 (0–0.2)	0.64
M1/M2 ratio, median (IQR)	0.60 (0.32–1.11)	0.62 (0.32–1.45)	0.88
No. of OCs per well, median (IQR)	61 (38–81)	68 (34–118)	0.52

*^a^Percentage within CD14-positive cells*.

*IQR, interquartile range, OCs, osteoclasts*.

### Positive Correlation between the M1/M2 Ratio and Greater Number of OCs

**Figure 2:**
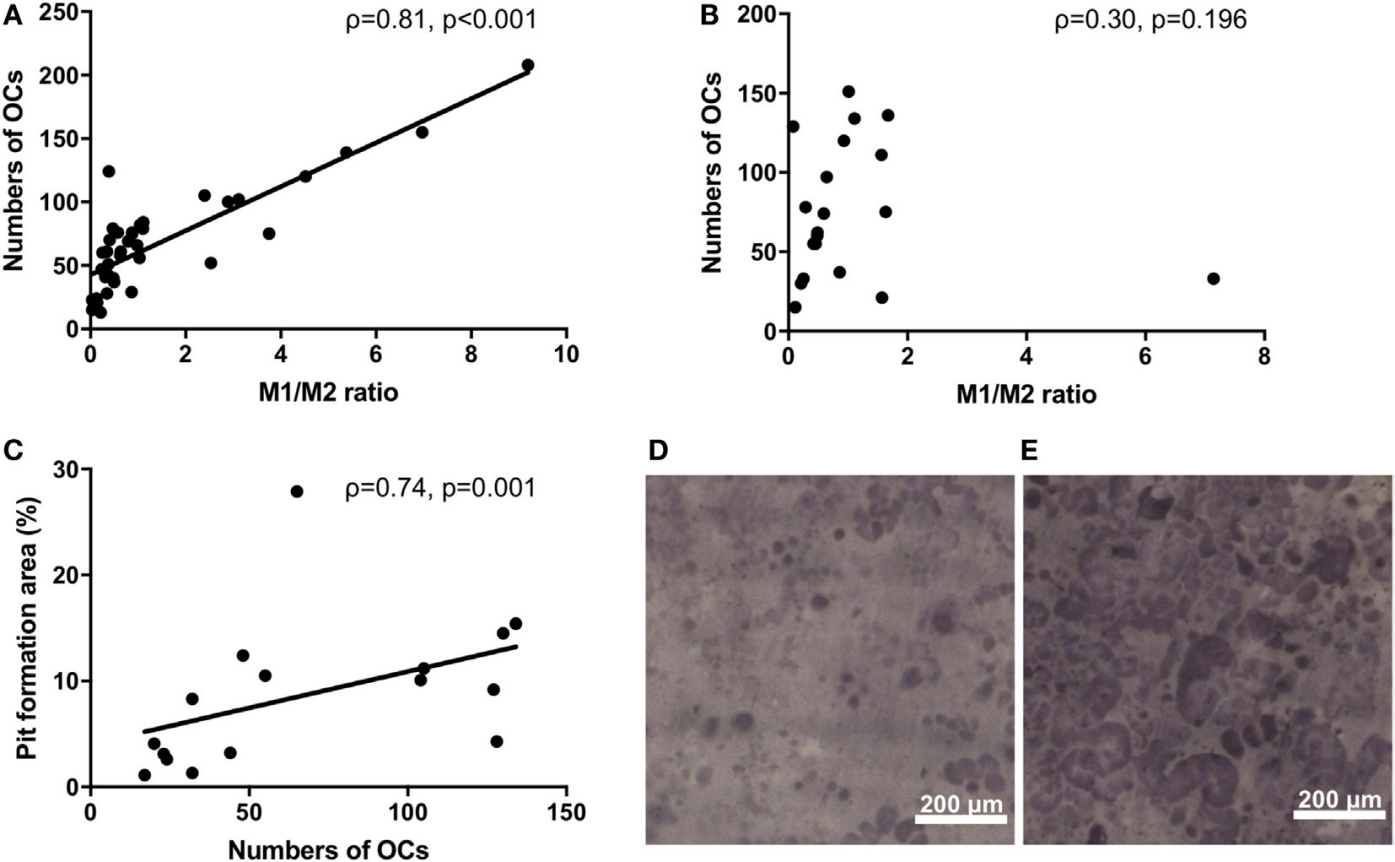
**(A)** The significantly positive correlation between the M1/M2 ratio and the number of osteoclasts in the rheumatoid arthritis patients (ρ = 0.81, *p* < 0.001). **(B)** No correlation between the M1/M2 ratio and the number of osteoclasts in the healthy donors. **(C)** The significantly positive correlation between the number of osteoclasts and the area percentage of the pit formation area (ρ = 0.74, *p* = 0.001). **(D)** Patients who had lower M1/M2 ratios and fewer numbers of osteoclasts had smaller resorbed areas. **(E)** Patients who had higher M1/2 ratios and greater numbers of osteoclasts had larger resorbed areas.

Monocytes are capable of differentiating into OCs. We next investigated the osteoclastogenic ability of M1/M2 monocytes. There was a significantly positive correlation between the M1/M2 ratio and the number of OCs in the RA patients (ρ = 0.81, *p* < 0.001) (Figure 2A). In contrast, there was no significant correlation between the M1/M2 ratio and the number of OCs in the healthy donors (ρ = 0.30, *p* = 0.196) (Figure 2B). The numbers of OCs *in vitro* were significantly correlated with the area percentage of the pit formation area (ρ = 0.74, *p* = 0.001) (Figure 2C), which demonstrated that the RA patients who had lower M1/M2 ratios and fewer OCs had smaller resorbed areas (Figure 2D) compared to the RA patients who had higher M1/2 ratios and greater numbers of OCs (Figure 2E).

### The ACPA-Positive RA Patients Had Higher M1/M2 Ratios and Greater Numbers of OCs

**Figure 3:**
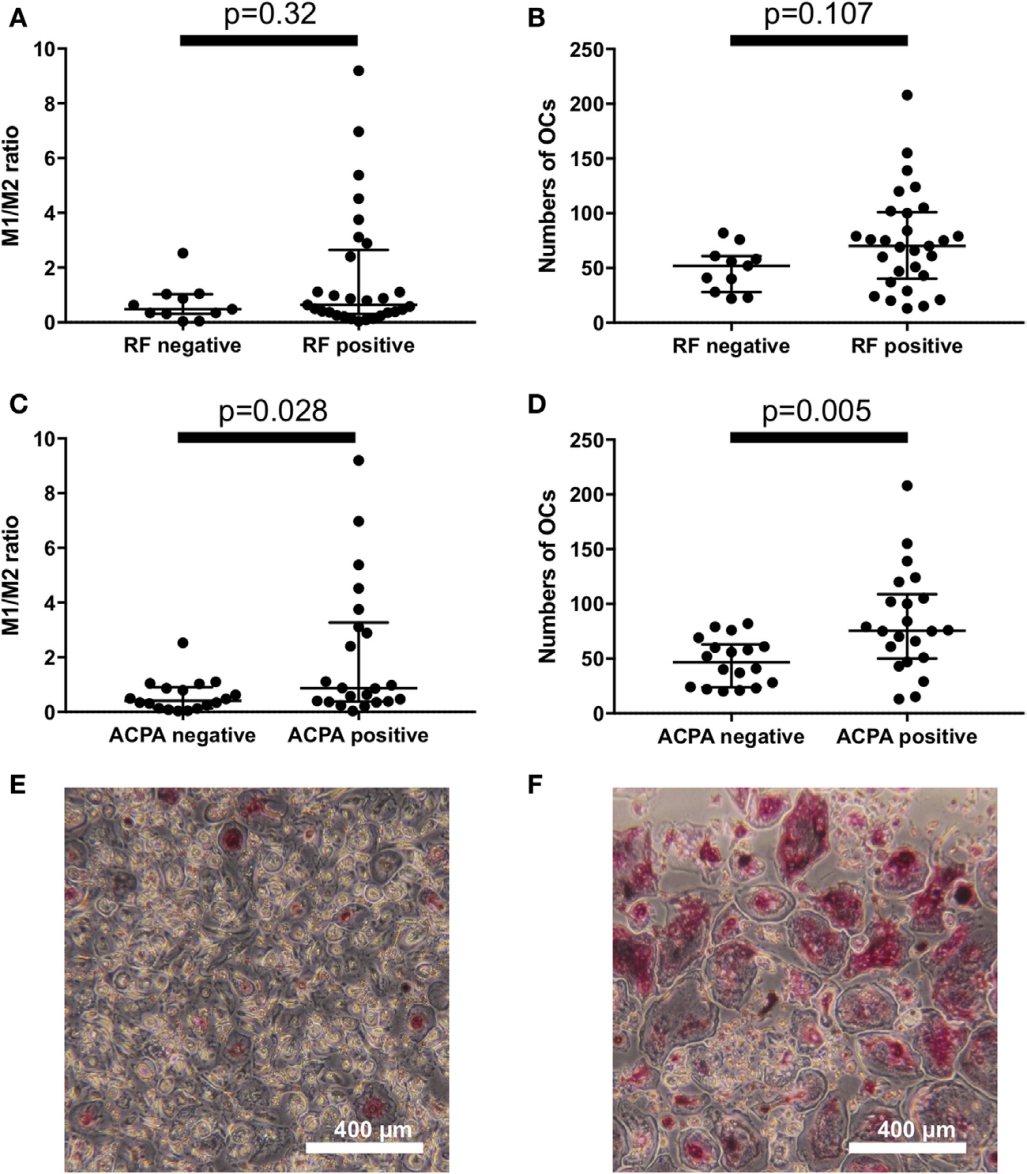
There were no significant differences between the rheumatoid factor (RF)-positive patients and RF-negative patients regarding **(A)** the M1/M2 ratio and **(B)** the number of osteoclasts. Anticitrullinated protein antibody (ACPA)-positive patients had **(C)** significantly higher M1/M2 ratios (0.87 vs. 0.41, *p* = 0.028) and **(D)** greater numbers of osteoclasts (76 vs. 47 per well, *p* = 0.005). **(E)** ACPA-negative patients had fewer osteoclasts *in vitro* compared to the ACPA-positive patients **(F)**.

To explore the factors that influence the M1/M2 subsets in RA, we analyzed the influence of RF/ACPA positivity on the M1/M2 subsets. There were no significant differences between the RF-positive RA patients and the RF-negative RA patients regarding the M1/M2 ratio and the number of OC (Figures 3A,B). In contrast, the ACPA-positive patients had significantly higher M1/M2 ratios (0.87 vs. 0.41, *p* = 0.028) (Figure 3C) and greater numbers of OCs (76 vs. 47 per well, *p* = 0.005) (Figure 3D). We show an ACPA-negative patient’s OCs *in vitro* of (Figure 3E) and those of an ACPA-positive patient (Figure 3F).

**Figure 4:**
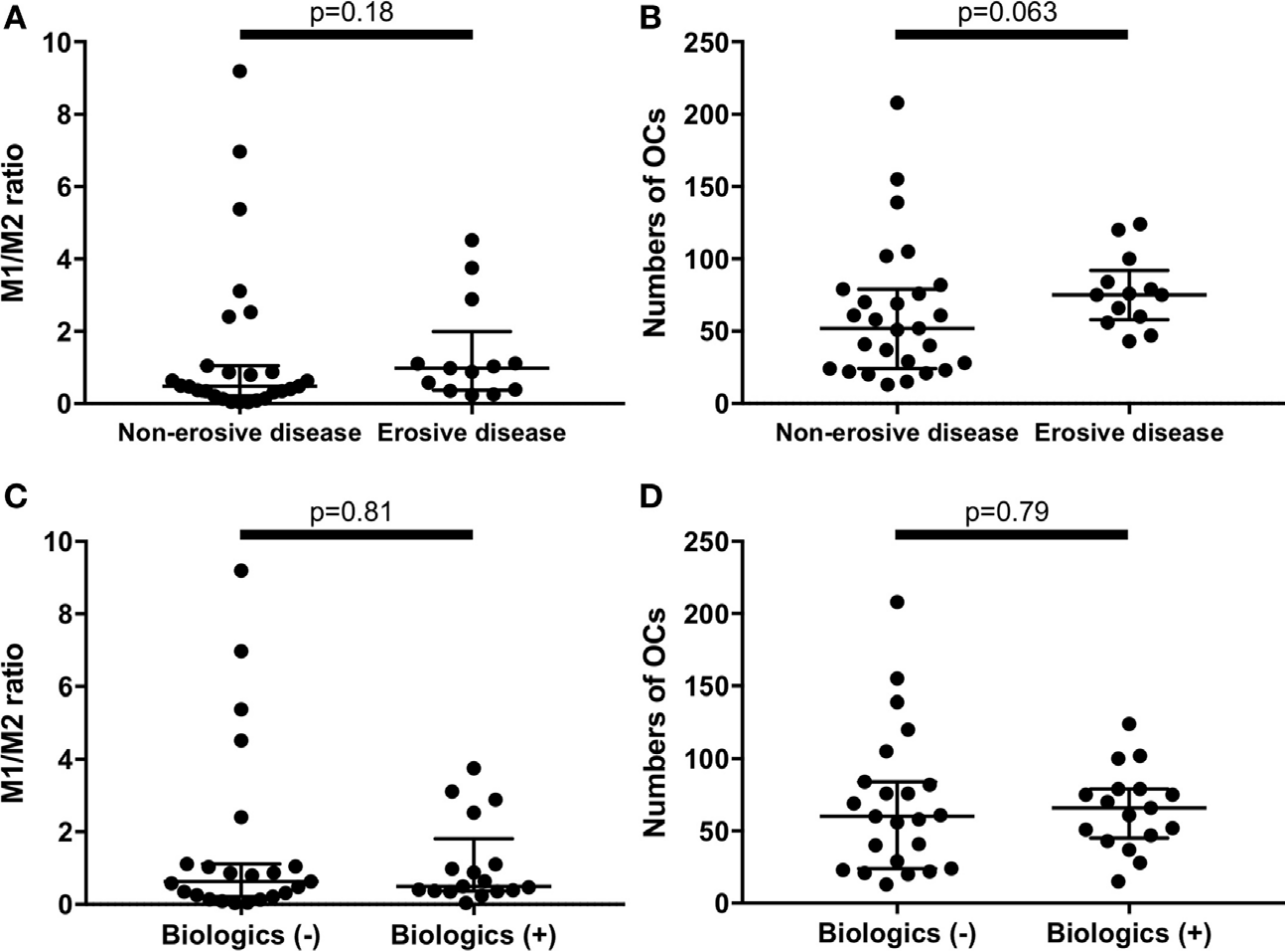
There were no significant differences between erosive rheumatoid arthritis (RA) patients at entry (*n* = 13) and the non-erosive RA patients at entry (*n* = 27) regarding **(A)** the M1/M2 ratio and **(B)** the numbers of osteoclasts. There were no significant differences between RA patients treated with biologics (*n* = 17) and RA patients treated without biologics (*n* = 23) regarding **(C)** the M1/M2 ratio and **(D)** the numbers of osteoclasts.

There were no significant differences between erosive RA patients at entry (*n* = 13) and the non-erosive RA patients at entry (*n* = 27) regarding the M1/M2 ratio and the number of OC (Figures 4A,B). In addition, there were no significant differences between RA patients treated with biologics (*n* = 17, 7 patients treated with TNF inhibitors, 5 patients treated with tocilizumab, and 5 patients treated with abatacept) and RA patients treated without biologics (*n* = 23) regarding the M1/M2 ratio and the number of OC (Figures 4C,D).

### The M1/M2 Imbalance Is a Strong Contribution Factor for Osteoclastogenesis

Anticitrullinated protein antibody is a well-known bone erosion promoting factor in RA (20). To elucidate which factor is crucial for osteoclastogenesis, we investigated factors that may contribute to the number of OCs by performing univariate and multivariable analyses (Table 3). In the univariate analysis, the positivities for RF and ACPA and the M1/M2 ratio significantly contributed to the numbers of OCs. However, the multivariable regression analysis revealed that the M1/M2 ratio was the sole significant factor contributing to the number of OCs (β-coefficient: 16.3, *p* < 0.0001).

**Table 3:** Multiple regression analysis of osteoclastogenesis.

Variable	Univariate analysis		Multivariable analysis	
	β-Coefficient (SE)	*p*-Value	β-Coefficient (SE)	*p*-Value
Age at entry	−0.399431 (0.244509)	0.1078	0.0188827 (0.215319)	0.9306
Sex (female to male)	3.170388 (5.547482)	0.5699		
Disease duration	0.0139225 (0.043056)	0.7482		
Patient visual analog scale	0.0264302 (0.261145)	0.9199		
Tender joint count	1.4795168 (1.214523)	0.2307		
Swelling joint count	2.4993797 (2.294888)	0.2830		
ESR	0.3104651 (0.275281)	0.2665		
CRP	7.5393985 (6.1201)	0.2256		
RF positivity	14.15476 (6.843849)	0.0455	3.206615 (4.857558)	0.5135
ACPA positivity	18.16667 (5.96073)	0.0042	4.292139 (4.690373)	0.3664
M1/M2 ratio	12.725356 (2.410721)	<0.0001	16.284653 (2.022948)	<0.0001

*ESR, erythrocyte sedimentation rate; CRP, C-reactive protein; RF, rheumatoid factor; ACPA, anticitrullinated protein antibody*.

### An M1/M2 Ratio >1 Is Associated with Higher ESR and CRP Values and Greater Numbers of OCs

**Figure 5:**
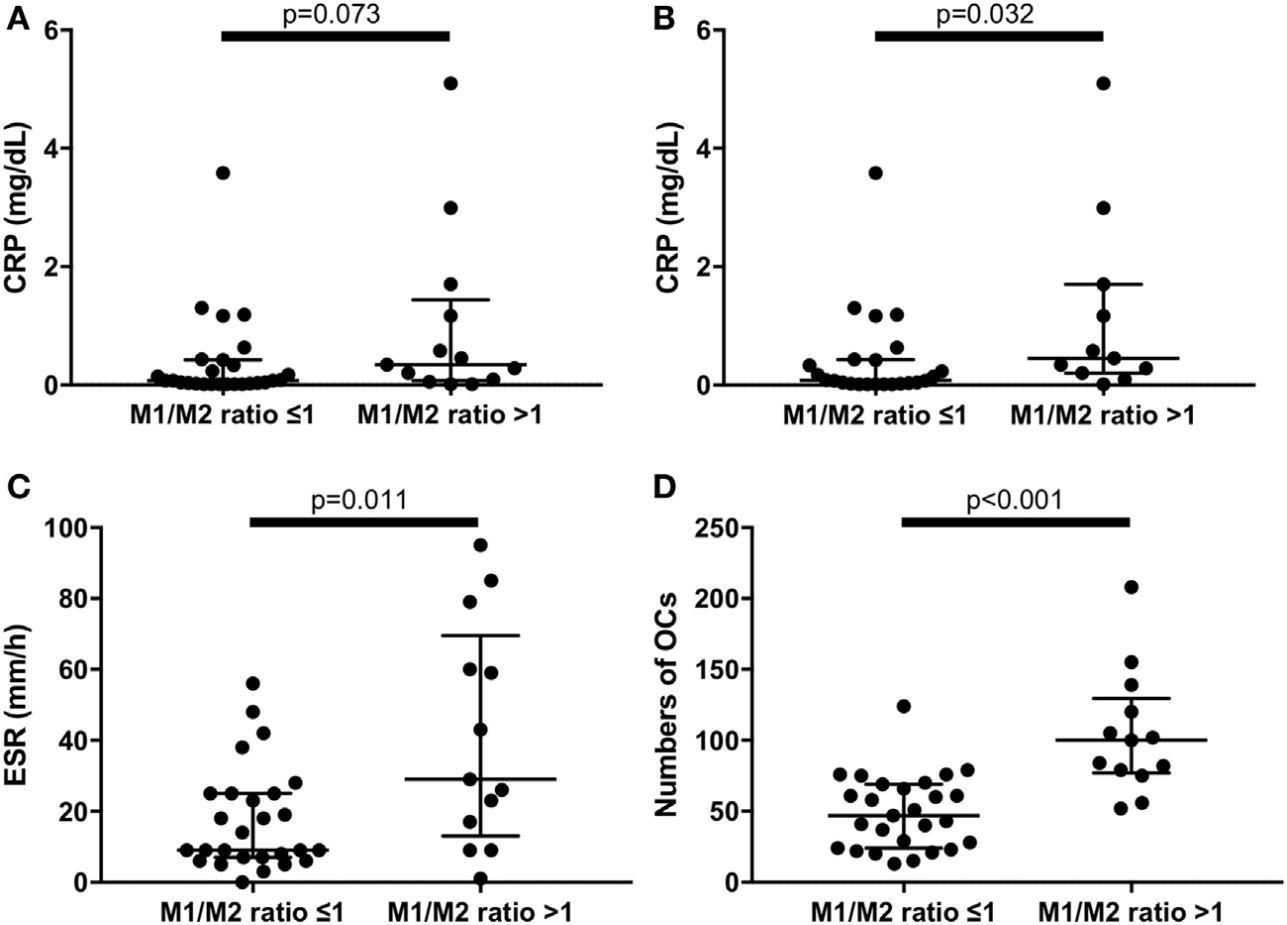
**(A)** There were no significant differences of C reactive protein (CRP) levels between M1/M2 ratio >1 group and M1/M2 ratio ≤1 group in the whole rheumatoid arthritis (RA) patients. **(B)** Excluding patients treated with tocilizumab, the M1/M2 ratio >1 group had significantly higher CRP levels (0.45 vs. 0.08 mg/dL, *p* = 0.032). The M1/M2 ratio >1 group had significantly **(C)** higher erythrocyte sedimentation rate (29 vs. 9 mm/1 h, *p* = 0.011) and **(D)** greater numbers of osteoclasts (100 vs. 47 cells per well, *p* < 0.001) in the whole RA patients.

An M1/M2 ratio >1 means that there are relatively more M1 than M2 monocytes. We divided the RA patients into two groups: those with an M1/M2 ratio >1 (*n* = 13) and those with an M1/M2 ratio ≤1 (*n* = 27). There were no significant differences of CRP levels between M1/M2 ratio >1 group and M1/M2 ratio ≤1 group (Figure 5A). Because tocilizumab is known to normalize CRP levels (21), we excluded patients treated with tocilizumab (*n* = 5) to evaluate CRP levels. As a result, the CRP levels were significantly higher in the M1/M2 ratio >1 group (0.08 vs. 0.45 mg/dL, *p* = 0.032) (Figure 5B). In addition, the M1/M2 ratio >1 group, which included patients treated with tocilizumab, had significantly higher ESR values (9 vs. 29 mm/1 h, *p* = 0.011) (Figure 5C). The M1/M2 ratio >1 patients had significantly higher numbers of OCs (47 vs. 100 cells per well, *p* < 0.001) (Figure 5D).

### High IL-6 Levels of Culture Supernatants of M1-Dominant Monocytes by LPS Stimulation

**Figure 6:**
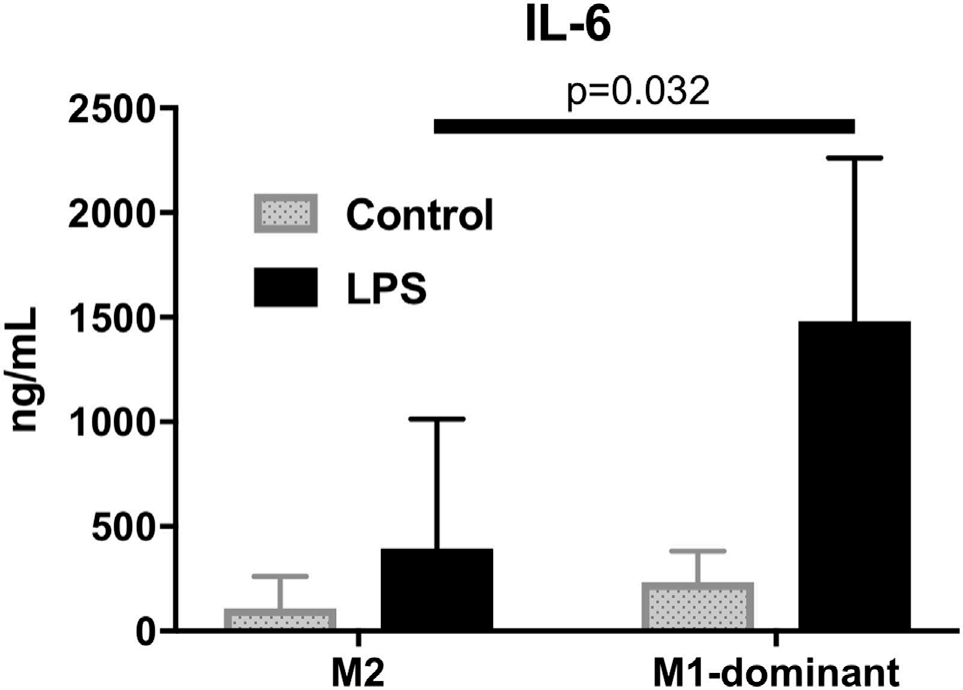
M1-dominant monocytes produced significantly more interleukin-6 than M2 monocytes did by stimulation of 100 ng/mL lipopolysaccharide (*n* = 5, healthy donors).

To analyze the function of M1/M2 monocytes in inflammation, we extracted M2 monocytes from whole monocytes and evaluated their cytokine production by measuring the IL-6 levels of the culture supernatants. The results demonstrated that the M1-domonant monocytes produced significantly more IL-6 than the M2 monocytes did (*n* = 5, *p* = 0.032, Figure 6).

## Discussion

This is the first study to show the possible relation of M1/M2 subsets with the pathogenesis of RA. We demonstrated that RA patients with positive ACPA had higher M1/M2 ratios and greater numbers of OCs. The patients with relatively more M1 monocytes in peripheral blood (M1/M2 ratio > 1) had higher ESR and CRP values and greater numbers of OCs.

To the best of our knowledge, a relationship between RA and M1/M2 monocyte subsets had not been reported prior to this study. However, research regarding CD68 and CD163 in inflammatory arthritis has been described. It was reported that CD68, one of the M1markers, positive synovial cells are mainly located in the sublining layer of rheumatoid synovial tissues, and the changes of the sublining of these cells were associated with clinical improvement by anti-rheumatic treatment (22). It was also reported that soluble CD163 in sera, one of the M2 markers, could be a diagnostic marker for macrophage activation syndrome in juvenile inflammatory arthritis (23). CD163 can bind and internalize hemoglobin–haptoglobin complexes, which leads to the strong anti-inflammatory effects by the release of IL-10 and carbon monoxide (24). CD163 may be produced to address overwhelming inflammatory reaction by macrophage activation syndrome in juvenile inflammatory arthritis.

There are two hypotheses regarding M1/M2 monocyte subsets and osteoclastogenesis. First, M1 monocytes may have higher osteoclastogenic differentiation ability than M2 monocytes. Second, higher M1/M2 ratios may create a proinflammatory environment that can induce more OC differentiation through some types of cytokines and chemokines, since M1-dominant monocytes produce excess amounts of IL-6, one of the most important osteoclastogenic cytokines in RA (25).

An association between ACPA, which predicts erosive disease (20), and high M1/M2 ratios was observed in the present study, but it appears likely that varying pathogenic mechanisms may lead to a final common pathway, resulting in macrophage activation as well as OC differentiation. In this regard, our multivariable analysis which revealed that a high M1/M2 ratio, not positivity of ACPA, was the sole contributing factor to osteoclastogenesis *in vitro* suggests that there may be another mechanism underlying the increase in the M1/M2 ratio that does not involve ACPA. One of the possible ACPA-independent mechanisms contributing to *in vitro* osteoclastogenesis might be the intrinsic characteristic of M1 monocytes and/or the inflammatory condition derived by M1 monocytes.

In the meantime, polyclonal ACPA and some of the monoclonal ACPAs are reported to enhance OC differentiation from monocytes of RA patients *in vitro* (26). It is also reported that ACPA immune complex could induce the monocytes’ production of inflammatory cytokines (27). Considering these results, one possible mechanism is that ACPA may bind to monocytes, creating an inflammatory environment and increasing the M1/M2 ratio, resulting in enhanced OC activation. However, this hypothesis cannot be supported by our multivariable analysis. Accordingly, the associations among the M1/M2 ratio, ACPA and osteoclastogenesis must be elucidated.

Our present RA patients with M1/M2 ratios >1 (indicating more M1 monocytes than M2 monocytes) had higher levels of ESR and CRP compared to the RA patients with M1/M2 ratios ≤1. Monocytes from preeclampsia patients with surface receptors characteristic of the M1 monocytes were reported to produce higher levels of proinflammatory cytokines such as higher TNF-α and IL-12p40/70 (28). These results as well as our finding that M1 monocytes produce more IL-6 support the idea that RA patients with M1/M2 ratios >1 had higher ESR and higher CRP through inflammatory cytokine networks.

Our study has some limitations. First, we should have used M1 monocytes instead of M1-dominant monocytes. However, we could not sort M1 monocytes by fluorescence-activated cell sorting because CD68 expresses in cytoplasm. A new method to obtain M1 monocytes without deconstruction of the cell membrane is needed. Second, we did not evaluate time courses of changes of radiographic bone erosions. Our data finding that a higher M1/M2 ratio results in more osteoclastogenesis *in vitro* should be confirmed clinically by radiographic findings of bone erosions over time.

In conclusion, our present findings demonstrated that the M1/M2 ratio is strongly correlated with the *in vitro* differentiation of OCs in patients with RA. M1 and M2 monocyte subsets may become a new target of treatments for RA to prevent progression of bone erosions.

## Ethics Statement

This study was performed in accordance with the Declaration of Helsinki and was approved by the Investigation and Ethics Committee at Nagasaki University Hospital (approval number: 12072361). Patients and healthy donors gave their informed consent to be subjected to the protocol.

## Author Contributions

SF drafted the manuscript and made substantial contributions to the concept and design with the assistance and supervision of NI. SF, NI, AT, TI, TS, MU, AN, YoH, YaH, TK, SYK, MT, KI, HN, TO, and AK treated the patients and collected the primary data. SF and NI performed the osteoclastogenesis *in vitro* and pit assays with the assistance of RM. SF and NI performed the ELISA. KK and KY performed the FACS analysis. SF performed the statistical analysis with the assistance of NI. NI, RM, KY, and AK critically revised the manuscript. AK supervised the entire study and gave final approval of the article. All authors read and approved the final manuscript.

## Conflict of Interest Statement

The authors declare that the research was conducted in the absence of any commercial or financial relationships that could be construed as a potential conflict of interest.
